# Animal source food consumption and anaemia among school adolescent girls in Silti District, Central Ethiopia: a public health perspective

**DOI:** 10.1017/jns.2024.76

**Published:** 2024-12-05

**Authors:** Shemsu Kedir, Kalkidan Hassen Abate, Bekri Mohammed, Kemal Lemnuro, Abdurezak Kemal, Sherif Khelil Geda, Zeyne Yassin, Musa Jemal, Beyene Wondafrash Ademe

**Affiliations:** 1 Department of Public Health, College of Medicine and Health Sciences, Werabe University, Werabe, Ethiopia; 2 Department of Nutrition and Dietetics, Institute of Health, Jimma University, Jimma, Ethiopia; 3 Department of Nutrition and Dietetics, Institute of Public Health, College of Medicine and Health Sciences, University of Gondar, Gondar, Ethiopia; 4 Department of Medicine, College of Medicine and Health Sciences, Werabe University, Werabe, Ethiopia; 5 Department of Health Behavior and Society, Institute of Health, Jimma University, Jimma, Ethiopia; 6 Department of Ophthalmology, Mehal Amba Primary Hospital, Mehal Amba, Ethiopia; 7 Department of Integrated Disease Surveillance Response, Regional Health Bureau, Werabe, Central Ethiopia

**Keywords:** Adolescent, Anaemia, Animal source food, Central Ethiopia, Silti District, ASF, Animal Source Food, AOR, Adjusted Odd Ratio, HFIAS, Household Food Insecurity Access Scale, FANTA, Food and Nutrition Technical Assistance, HDDS, Household Dietary Diversity Score

## Abstract

Animal source foods (ASFs) are diverse and heterogeneous, including unprocessed red meat, processed meat, poultry, eggs, seafood, milk, cheese, and yoghurt. It is essential for preventing malnutrition and its consequences among vulnerable populations, including infants, young children, adolescents, women of reproductive age, and pregnant and lactating women. Understanding the intakes of ASF among adolescents and associated factors is critical for establishing priorities to promote its consumption and enhance growth and development during this period. Consequently, the purpose of the current study is to examine the current levels of not consuming ASF among school-aged adolescent girls and the relationship between anaemia and not consuming ASF. A facility-based cross-sectional study design was conducted among 516 school adolescent girls in Silti District, Central Ethiopia, spanning from October 2 to 20, 2023. Data were entered into Epi-data 3.1 and exported to SPSS version 25 for further analysis. Adjusted odds ratios, along with their corresponding 95% confidence intervals, were calculated to examine the association between not consuming ASF and anaemia status after adjusting for age, residence, menstrual status, and morbidity. Statistical significance was defined as a p-value less than 0.05 in the final model. In this study, the prevalence of not consuming any form of ASF was 51.1% (95 CI: 44.6%–53.2%) and the prevalence of anaemia was 29% (95% CI: 25.2, 33.3). Moreover, unlikely consumption of ASF was significantly associated with anaemia (AOR = 3.2, 95% CI:2, 5.1) after adjusting for age, place of residence, experience of morbidity symptoms and menstrual status. The current study found a significant prevalence of adolescent girls in the area not consuming ASF while attending school. Moreover, the prevalence of anaemia was moderate. Unlikely consumption of ASF was significantly associated with anaemia. Hence, enhancing ASF consumption is one of the key actions to fight against anaemia.

## Introduction

Adolescence, the period of rapid physical, cognitive, and emotional growth that separates childhood from adulthood lays the foundation for lifelong good health and well-being.^(1)^ Thus nutrition during adolescence affects linear growth, body composition, brain growth and development, and immune function, as exemplified by the increased rate of anaemia, as a consequence of poor diet during adolescence.^(2,3)^

Animal source foods (ASFs) are diverse and heterogeneous, including unprocessed red meat, processed meat, poultry, eggs, seafood, milk, cheese, and yoghurt. These foods often contain high and bioavailable contents of important nutrients, for example, vitamin A, folic acid, calcium, iodine, iron, zinc, essential fatty acids, and protein,^(4)^ are the only natural dietary sources of vitamins B12 and D, and can have higher levels of some nutrients (eg, vitamin A, folate, vitamin B12, calcium, iron, and zinc) than plant source foods.^(5–8)^ These characteristics make ASF useful for improving nutrition in vulnerable populations such as infants, young children, adolescents, women of reproductive age, pregnant and lactating women, and older adults, as well as very poor communities in low-income and middle-income countries.^(9–11)^

ASFs are vital components of healthy diets, helping to reduce the risk of under nutrition, including anaemia, particularly in vulnerable populations like adolescents.^(12)^ They provide high-quality proteins that contain all essential amino acids in the necessary quantities and forms for human health. ASFs are energy-dense and rich in bioavailable micronutrients such as calcium, iron, zinc, vitamin A, vitamin B12, and riboflavin, which are crucial for addressing global food insecurity. On top of that, compared to foods derived from plants, they have lower levels of anti-nutritional factors.^(13)^

Consumption of such foods has been found to increase dietary diversity and quality, which in turn strongly contribute to improved nutritional results. It is thought that consuming ASF, even in small amounts, can provide the body with essential micronutrients and protein,^(14,15)^ despite social, economic, and cultural barriers related to its intake.^(14)^ Evidence additionally suggests that inadequate or poor ASF intake throughout life is strongly linked to poor physical growth, impaired cognition, morbidity, and mortality.^(15)^ Moreover, the high bioavailability of ASFs’ nutrients — especially those that impact anaemia — makes them particularly advantageous. Vitamin A (meat, eggs), vitamin B12 (meat, eggs, milk), and heme iron (high in beef, other red meats, and organ meats) may all contribute to anaemia if they are deficient.^(16)^

Worldwide, reports indicated that in 2018 the average daily intake of ASF among adolescents (aged 10 to 19) was approximately 2.5 servings per day. Trailing only to Southern Asia in terms of daily consumption, the report indicated that Sub-Saharan African adolescents are the least consumers of ASF, at roughly a serving per day.^(3)^

Ethiopia is known to possess one of the world’s largest populations of livestock.^(17)^ Nonetheless, there is a reported low intake of meat, and fish. Given that ASF is viewed as an enjoyment diet rather than an essential component of the regular family diet, it is frequently consumed during extra special family or public events in settings that are predominately rural.^(18)^ The consumption of milk and meat in Ethiopia is very low, even compared with other African countries.^(19)^

Ethiopia has been executing the food-based dietary guideline,^(20)^ the National Nutrition Program,^(21)^ recently endorsed a Food and Nutrition Policy,^(22)^ and applied a National Food and Nutrition Strategy^(23)^ as avenues to address the issues of poor dietary behaviour, food consumption, and the consequent under-nutrition. Regrettably, malnutrition, particularly in adolescents, is a concerning issue in Ethiopia due to inadequate food consumption and poor dietary habits.^(24)^

To the best of the researchers’ knowledge, there is no ample evidence of adolescent ASF consumption and its associated factors, and evidence of the relationship between ASF consumption and anaemia is also lacking in Ethiopia. Understanding the intakes of ASF among adolescents and associated factors is critical for establishing priorities to promote its consumption, enhance growth and development during this period, and promote health in adulthood. Consequently, the current study aims to examine the current levels of not consuming ASF among school-aged adolescents and the association between anaemia and not consuming ASF.

## Methods and materials

### Study design, setting, and sampling

This facility-based cross-sectional study was conducted in Silti District, Central Ethiopia, from October 2 to 20, 2023. The district predominantly relies on cereal-based agriculture and practices mixed crop-livestock production, with inhabitants residing in permanent settlements. It encompasses 25 Kebele, including three designated as urban areas. The projected population for 2022 is 151,573, with 21,725 school adolescents, among whom 10,669 are girls, and 8,721 attend primary schools. Within the study area, there are 18 rural and 8 urban governmental schools. Of these, eight schools were randomly selected considering the proportion of residents (2 from urban and 6 from Rural).

The final sample size of 516 was determined using the single population proportion formula following 20% ASF consumption^(19)^ from the previous study with a design effect of 2 and 5% non-response rate. A multi-stage sampling method was utilised to select eligible adolescents for the study. Initially, schools were randomly chosen from the study area. The allocation of the sample size at each site was determined by considering the relative proportion of the selected study population at that site.

In this study, the source population was all adolescent students who resided in the district and the study population was those randomly selected schools that fulfilled the inclusion criteria. Eligibility criteria included students aged 10–19 years enrolled from grades 4–12. Students with severe illnesses or an inability to communicate were excluded from the study.

### Data collection methods

Data collection employed structured questionnaires initially developed in English, then translated into Amharic and Siltigna (Local) languages. The translated versions were back-translated into English for validation. Eight interviewers and two supervisors were selected based on their proficiency in both Amharic and Siltigna (Local) languages, along with relevant prior experience. Their educational backgrounds were Degree levels.

The wealth index and household food security questionnaire were administered by the household head, whereas the adolescent questionnaire encompassed inquiries regarding education, ASF consumption, health, nutrition, anthropometric measurements, and laboratory tests. Following the household head interview, adolescents were interviewed subsequently.

Prior to pre-testing the forms, interviewers and supervisors underwent two days of rigorous training. The questionnaires underwent pre-testing (5%) in the participants out of the study area at Balo-Kariso Primary School to identify and rectify any concerns related to content, wording, and formatting. Supervisors oversaw field procedures and conducted daily reviews of completed questionnaires to ensure the accuracy of the collected data.

## Measurements and operational definition

**Consumption of ASF:** Participants were queried about the types of foods consumed by adolescents in the last week preceding the survey. The questionnaire included eggs, fish, yoghurt, cheese, milk, meat (such as beef, poultry, lamb, and other unspecified meats), and organ meats (e.g., liver) as ASFs.^(19)^ The study’s outcome variable was the consumption of ASFs by school adolescents, categorised as either ‘0’ (not consuming ASF) or ‘1’ (ASF consumption). Any intake of the ASFs listed above, regardless of amount or type, was considered ASF consumption.^(19)^

**Wealth index:** Socioeconomic status was determined through a two-step process. First, participants were surveyed about household ownership of fixed assets, with a score of one assigned to owners and zero to non-owners. Second, principal component analysis was used to create a wealth index. The wealth index was then categorised into tertiles: low, middle, and high wealth.

**The common morbidity frequency symptoms**: In this study, the common morbidity frequency symptoms observed were diarrhoea, cough, and fever. A history of these symptoms over the last two weeks was collected, and each symptom was assigned a score based on its presence or absence: Fever, diarrhoea, and cough were scored as 1 if present and 0 if absent. The Morbidity Frequency Score was calculated as the sum of fever, cough, and diarrhoea. Individuals with a morbidity frequency score of 0 experienced none of the specified symptoms, while scores of 1, 2, or 3 indicated varying levels of morbidity.

**Household Food Security status:** Household Food Insecurity Access Scale (HFIAS), a validated tool created by the Food and Nutrition Technical Assistance programme, is used to assess the food security status of households in our study. HFIAS consists of nine occurrence questions and nine follow-up questions aimed at assessing the frequency of food insecurity occurrences, offering valuable information on the intensity and regularity of challenges in accessing food faced by households. These sets of questions have been utilised in different countries and have proven to be effective in distinguishing between households that are food secure and those that are food insecure, regardless of cultural differences.^(25)^ Each household’s HFIAs score is calculated by summing up the codes for each frequency or occurrence question. Instances where the response to occurrence questions is ‘No’ are coded as ‘0’ before summing the frequency of occurrence codes. Scores range from 0 (indicating no reported occurrences of food insecurity) to 27 (when all nine frequencies of occurrence questions receive a response code of 3). Higher scores indicate greater food insecurity, while lower scores suggest less food insecurity in the household.^(25,26)^

**Menstruation Status:** Respondents were asked if they had ever experienced menstruation, with options for yes or no responses.

### Haemoglobin analysis

Adolescent school participants underwent blood haemoglobin concentration assessments. A trained laboratory technician collected 5 ml venous blood samples using sterile syringes. Samples were stored in a cold chain system and transported daily to Werabe Comprehensive Specialized Hospital. Anaemia prevalence was determined using Sysmex-5 Differential haematology Machine, with haemoglobin concentrations adjusted for altitude, sex, and age using established formulas to account for reduced oxygen saturation at high altitudes.^(27)^

Anaemia is characterised by low blood haemoglobin concentration (<12 g/dl), with consideration for age, sex, and altitude, while moderate anaemia denotes haemoglobin levels between 7 and 12 g/dl, and severe anemia is defined by haemoglobin levels <7 g/dl.^(27)^

## Data analysis

Data were entered into EPI-Data version 3.1 using a double-entry process. The data were then rigorously cleaned, coded, and examined for missing values and outliers to ensure data integrity for subsequent analysis. Finally, the data were exported to SPSS version 25 statistical software for analysis.

Bivariable and multivariable binary logistic regression analyses were conducted to evaluate the relationship between each independent variable and the outcomes of interest. Variables with a p-value less than 0.2 in the bivariate analyses were included in the final multivariable model to control for potential confounders. Additionally, multicollinearity diagnostics were performed to assess the independence of the included variables. Adjusted odds ratios, along with their corresponding 95% confidence intervals, were calculated to examine the association between not consuming ASF and anaemia status adjusted for age, residence, menstrual status, and morbidity. Statistical significance was defined as a p-value less than 0.05 in the final model.

## Ethical consideration

This study’s methodologies adhered to the ethical principles outlined in the Declaration of Helsinki for medical research involving human subjects. An ethical approval letter was obtained from the Institutional Review Board (IRB) of Jimma University. An official letter of cooperation was written to Silte zones and the district education office. Participants remained anonymous in all reports, with enrolment being voluntary and withdrawal permitted at any stage. The study details were verbally explained in accordance with the information sheet, and consent and assent were obtained through signatures or thumbprints from mothers/caregivers and adolescents, respectively.

## Result

In the present study, among the total of 516 participants, 489 participated with a response rate of 94.8%. More than half (58.3%) of the participant’s age were in the middle adolescent category. Almost sixty per cent (59.5%) of the participants were rural residents. Thirty-two per cent of participants were on a low wealth index. Furthermore, forty-two per cent of study participants were food insecure households (Table 1).

**Table 1.** Socio-demographic characteristics of school adolescent girls in Silti District, 2023

Variable	Category	Frequency
Age (mean/SD)	–	14.77 ± 1.65
Age	Early Adolescent (10–13)	23.5%
	Middle Adolescent (14–16)	58.3%
	Late Adolescent (17–19)	18.2%
Family size	≤5	36.8%
	≥6	63.2%
Residence	Urban	40.5%
	Rural	59.5%
Wealth Index	Low	32.5%
	medium	39.1%
	high	28.4%
HFIAS	Secure	58.1%
	Insecure	41.9

### ASF consumption status disaggregated by HFIAS and wealth status

**Figure 1.**
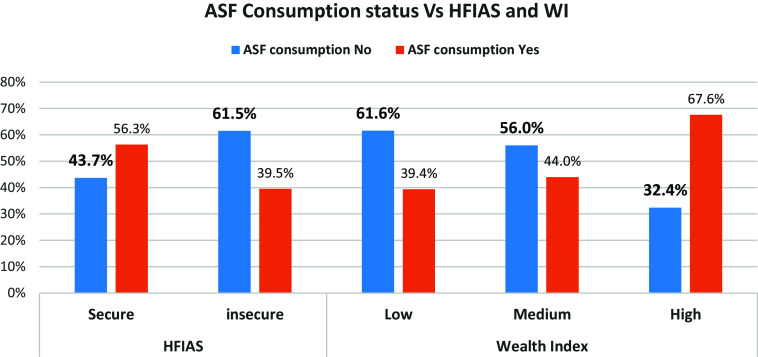
Animal source food consumption status with wealth index and Household Food Insecurity Access Scale among school adolescent girls in Silti District, Central Ethiopia, 2023.

More than sixty per cent of food insecure and low-wealth quintile participants didn’t consume ASF (Fig. 1).

### Anaemia status disaggregated by ASF consumption status

**Figure 2.**
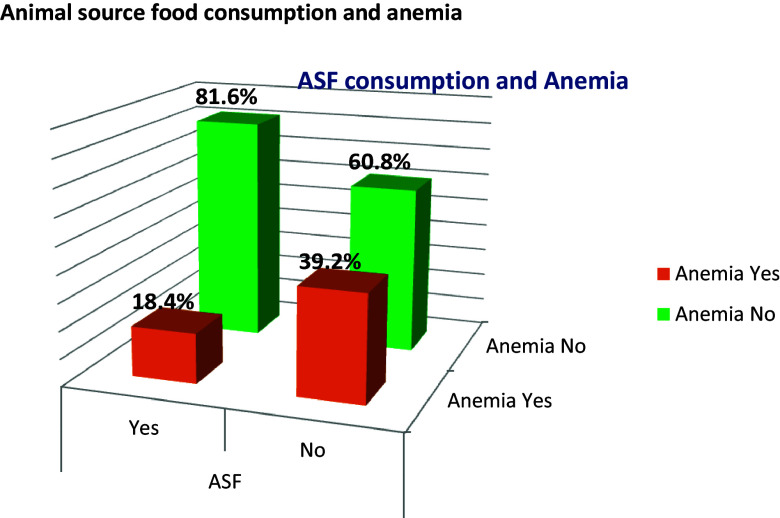
Animal source food consumption status and anaemia among school adolescent girls in Silti District, Central Ethiopia, 2023.

Figure 2 indicated that the proportion of anaemia among not consumed ASF participants (39.2%) was higher than anaemia among those who consumed ASF (18.4%).

### ASF consumption and anaemia Status

**Figure 3.**
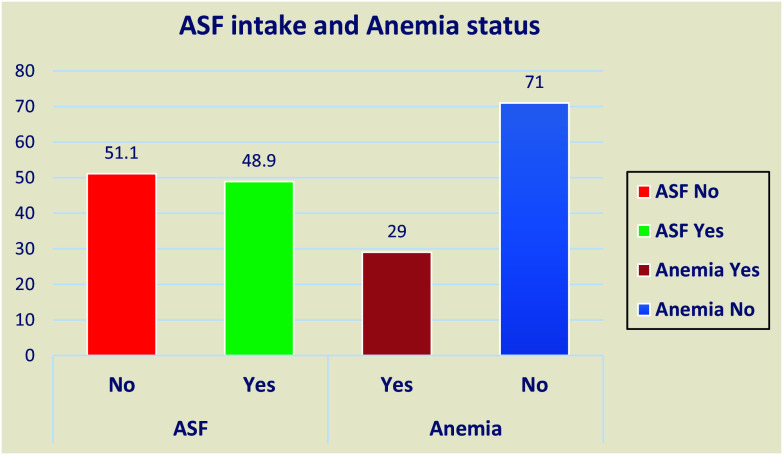
Animal source food intake status among adolescent girls in Silti District, Central Ethiopia, 2023.

In this study, the prevalence of not consuming any form of ASF was 51.1% (95 CI: 44.6%–53.2%) and the prevalence of anaemia was 29% (95% CI: 25.2, 33.3) (Fig. 3).

### Association of anaemia status and ASF consumption

After adjusting for age, place of residence, HFIAS, wealth index, experience of morbidity symptoms and menstrual status, ASF consumption was significantly associated with anaemia. Those who were not consuming ASF were 3.2 times more likely to have anaemia compared to their counterpart (AOR = 3.2, 95% CI: 2, 5.1) (Table 2).

**Table 2.** Final model for association of anaemia status and animal source food (ASF) consumption

Variable	COR (p-value)	AOR (p-value)	AOR 95%CI	
			Lower boundary	Upper boundary
Anemia ←				
ASF non consumer^**^	2.8 (<0.001)	3.22 (0.0001)	2.03	5.10
Constant				

** **N.B**. this association ASF consumption and anaemia status is adjusted for age, residence, menstrual status and morbidity symptom frequency.

### ASF consumption and associated variables

After adjusting for age, residence, and family size, ASF intake was strongly associated with HFIAS and wealth status. Food-insecure adolescent girls were almost twice as unlikely to consume ASF compared to their food-secure counterparts (AOR = 1.97, 95% CI: 1.3, 2.88). Moreover, participants in low wealth tertile were 3.2 times more unlikely to consume ASF compared to the highest-wealth tertile (AOR = 3.2, 95% CI: 1.9, 5.2) (Table 3).

**Table 3.** Multiple binary logistic regression of animal source food (ASF) and associated variables

					95% C.I.for EXP(B)	
Variables in the equation		B	Sig.	AOR	Lower	Upper
ASF consumption ←						
Age in years	Early adolescence (10–13)	−0.031	0.917	.970	.543	1.732
	Middle adolescence (14–16)	−0.025	0.923	.976	.591	1.611
	Late adolescence (17–19)					
Family Size	<5			1		
	≥5	0.227	0.251	1.255	.851	1.851
HFIAS	Insecure	0.680	0.001	1.973	1.348	2.887
	Secure			1		
Wealth Status	Low	1.164	0.0001	3.204	1.971	5.208
	Middle	0.966	0.0001	2.628	1.652	4.178
	Highest			1		
Residence	Rural	−0.223	0.253	0.800	0.546	1.173
	Urban			1		

## Discussion

The current investigation revealed a high prevalence of not consuming ASF among adolescent girls attending schools within the study area.

In this study, a notable prevalence of non-consumption of ASF was observed. This observation aligns with existing literature highlighting that adolescents in Sub-Saharan Africa exhibit notably low consumption of ASF, typically averaging around one serving per day.^(3)^ In addition to this, empirical evidence from Ethiopia indicates a strikingly low consumption of milk and meat products.^(19,28)^ Conversely, cereals served as the predominant food group consumed by a significant proportion (96%) of the population, while fish, eggs, and fruits were identified as the least consumed food groups.^(15)^ This may be due to that ASF is viewed as an enjoyment diet rather than an essential component of the regular family diet particularly for infant, adolescent, pregnant and lactating women, it is frequently consumed during extra special family or public events in settings that are predominately rural.^(18)^

Furthermore, the results of the current study underscored that individuals who did not consume ASF were three times more likely to experience anaemia after the adjustment for confounding factors such as age, place of residence, and family size. This significant association is consistent with a study conducted in the Aw-Barre refugee camp in the Somali regional state of Southeast Ethiopia,^(29)^ which found that individuals who consumed heme iron food sources less than once a month had a higher risk of developing anaemia compared to those who consumed them more than twice a week. Additionally, a study in Indonesia also supported this finding by demonstrating a significant relationship between protein intake and anaemia.^(30)^

One possible explanation for these associations is that proteins play a crucial role in haemoglobin synthesis, which is essential for preventing anaemia. This aligns with the findings of a study conducted in Japan, which suggested that higher protein intake may help reduce anaemia in females.^(31)^ Therefore, the link between consuming ASF, protein intake, and the risk of anaemia highlights the importance of including these nutrient-rich foods in the diet to support overall health and prevent nutritional deficiencies.

Nonetheless, it was observed that adolescents belonging to lower and middle wealth quantiles, as well as those residing in food insecure households, exhibited a significant association with unlikely to consume ASF. Specifically, households experiencing food insecurity were twice as unlikely to consume ASF compared to food-secure households. The results of this study align with previous research conducted in Ethiopia, which highlighted a notable disparity in ASF consumption between households categorised by their Household Dietary Diversity Score (HDDS).^(15)^ The study indicated that ASFs were more frequently included in the diets of households with higher HDDS scores, reflecting a greater variety and quality of food consumed compared to households with lower HDDS scores. This disparity underscores the critical role of household food security in shaping dietary patterns and access to nutrient-rich foods, particularly ASFs, which are essential sources of protein, vitamins, and minerals for overall health and well-being.^(15)^

Moreover, the results of this study highlighted that participants in the lower and middle wealth quintiles were unlikely to consume ASF. This observation aligns with a previous study conducted in rural Bangladesh, suggesting a consistent trend.^(15,32)^ One possible explanation for this pattern could be attributed to the economic constraints faced by families in these wealth categories. The cost of animal source foods tends to be higher compared to plant-based alternatives, making them less accessible to households with limited financial resources. As a result, individuals from lower and middle wealth quintiles may be more likely to abstain from consuming ASFs due to affordability issues.

### Strength and limitations

To the best of the investigator’s knowledge, this study represents the pioneering research in Ethiopia shedding light on those not consuming animal source foods (ASF) among adolescent girls. However, it is important to acknowledge certain limitations that may impact the interpretation of the findings. The inherent constraints of a cross-sectional study design pose limitations on the ability to establish causal relationships and determine temporality between variables. Furthermore, this study only focused on public schools, haemoglobin estimation, without considering other important haematological parameters. Additionally, the assessment of fever, cough and diarrhoea was solely based on participants’ recall history over the past two weeks, rather than laboratory-based testing, which could potentially compromise the accuracy and reliability of the data. These limitations underscore the need for further research with more comprehensive methodologies to provide a more robust understanding of the relationship between not consuming ASF, anaemia, and other related factors among adolescent girls in Ethiopia.

### Conclusion and recommendation

The current investigation revealed a high prevalence of not consuming ASF among adolescent girls attending schools within the study area. Moreover, the prevalence of anaemia was moderate. Fortunately, in Ethiopia school feeding programmes need to incorporate ASF into daily meals to mitigate malnutrition including anaemia. Launch educational campaigns in schools and communities to raise awareness about the importance of ASFs in the diet, particularly for adolescent girls.

## Data Availability

All relevant data are available in the hands of corresponding authors with a reasonable request.
